# Loss‐of‐function variants in RNA binding motif protein X‐linked induce neuronal defects contributing to amyotrophic lateral sclerosis pathogenesis

**DOI:** 10.1002/mco2.712

**Published:** 2024-09-10

**Authors:** Di He, Xinyi He, Dongchao Shen, Liyang Liu, Xunzhe Yang, Meng Hao, Yi Wang, Yi Li, Qing Liu, Mingsheng Liu, Jiucun Wang, Xue Zhang, Liying Cui

**Affiliations:** ^1^ Department of Neurology Beijing Tiantan Hospital, Capital Medical University Beijing China; ^2^ Department of Neurology Peking Union Medical College Hospital (PUMCH), Chinese Academy of Medical Sciences and Peking Union Medical College Beijing China; ^3^ State Key Laboratory of Genetic Engineering, Collaborative Innovation Center for Genetics and Development, School of Life Sciences and Human Phenome Institute Fudan University Shanghai China; ^4^ McKusick‐Zhang Center for Genetic Medicine, State Key Laboratory of Medical Molecular Biology Institute of Basic Medical Sciences Chinese Academy of Medical Sciences, School of Basic Medicine, Peking Union Medical College Beijing China; ^5^ Research Unit of Dissecting the Population Genetics and Developing New Technologies for Treatment and Prevention of Skin Phenotypes and Dermatological Diseases Chinese Academy of Medical Sciences (2019RU058) Shanghai China; ^6^ Neuroscience Center Chinese Academy of Medical Sciences and Peking Union Medical College (CAMS) Beijing China

**Keywords:** ALS, m6A modification, RBMX, single‐cell sequencing, whole‐exome sequencing

## Abstract

Despite being one of the most prevalent RNA modifications, the role of N6‐methyladenosine (m6A) in amyotrophic lateral sclerosis (ALS) remains ambiguous. In this investigation, we explore the contribution of genetic defects of m6A‐related genes to ALS pathogenesis. We scrutinized the mutation landscape of m6A genes through a comprehensive analysis of whole‐exome sequencing cohorts, encompassing 508 ALS patients and 1660 population‐matched controls. Our findings reveal a noteworthy enrichment of RNA binding motif protein X‐linked (*RBMX*) variants among ALS patients, with a significant correlation between pathogenic m6A variants and adverse clinical outcomes. Furthermore, *Rbmx* knockdown in NSC‐34 cells overexpressing mutant TDP43^Q331K^ results in cell death mediated by an augmented p53 response. Similarly, *RBMX* knockdown in ALS motor neurons derived from induced pluripotent stem cells (iPSCs) manifests morphological defects and activation of the p53 pathway. Transcriptional analysis using publicly available single‐cell sequencing data from the primary motor cortex indicates that RBMX‐regulated genes selectively influence excitatory neurons and exhibit enrichment in ALS‐implicated pathways. Through integrated analyses, our study underscores the emerging roles played by *RBMX* in ALS, suggesting a potential nexus between the disease and dysregulated m6A‐mediated mRNA metabolism.

## Graphical abstract

The dysregulation of m6A modification has gained recognition as a crucial factor in the development of amyotrophic lateral sclerosis (ALS). Among the m6A reader proteins, RNA binding motif protein X‐linked (RBMX) stands out with a notable enrichment of variants in ALS patients, and the presence of pathogenic RBMX variants is associated with a faster disease progression. In vitro experiments have provided evidence that reducing RBMX levels can result in neuronal defects. Additionally, bioinformatic analyses have supported these findings by revealing that RBMX‐associated genes specifically impact excitatory neurons. Furthermore, these genes are involved in the regulation of pathways and genes associated with neurodegeneration and RNA metabolism, underscoring the relevance of RBMX in ALS pathogenesis.

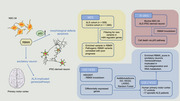

## 1 INTRODUCTION

Amyotrophic lateral sclerosis (ALS) stands as a progressive neurodegenerative disorder impacting both upper and lower motor neurons.^1^ The clinical manifestation of ALS exhibits considerable variability, with a majority of patients advancing to respiratory failure and succumbing to mortality within 5 years post disease onset.^2^, ^3^ Despite the escalating identification of novel disease‐associated mutations in familial cases, the etiology and genetic underpinnings of ALS remain only partially elucidated, particularly in cases devoid of a positive family history.^4^, ^5^, ^6^ Nevertheless, a pivotal pathological pathway shared among individuals with ALS involves dysregulated RNA metabolism, with several mutations implicated in the disease identified within genes that encode RNA‐binding proteins (RBPs), including *TARDBP*, *FUS*, *HNRNPA1*, and *HNRNPA2B1*.^7^ It is noteworthy that some of these RBPs are engaged in the regulation of mRNA metabolism and splicing through their recognition of the N6‐methyladenosine (m6A) RNA modification.^8^, ^9^, ^10^ These observations imply the probable involvement of dysregulated m6A modification in the pathophysiology of ALS.

As the most prevalent modification of eukaryotic mRNAs, the dynamic m6A modification exerts influence over a diverse array of biological processes (BPs), including mRNA maturation, stability, export, and translation efficiency.^11^, ^12^ Post‐transcriptionally, m6A modification is introduced, removed, and recognized by m6A methyltransferases, m6A demethylases, and m6A‐specific binding proteins, respectively.^13^, ^14^ The dysregulation of m6A modification has been associated with anomalies in tissue development, tumorigenesis, and immunity,^15^, ^16^, ^17^ and it is now gaining acknowledgment as a potential regulatory mechanism governing gene expression in the context of ALS. In an m6A quantitative trait loci (QTL) study, which disclosed a notable enrichment of m6A QTLs in ALS brain tissues, the authors posited *TARDBP* as a promising candidate m6A reader gene.^18^ Furthermore, a single‐cell profiling study of the human primary motor cortex revealed that several hub genes within ALS‐specific modules, as identified by weighted gene coexpression network analysis (WGCNA), were intricately involved in m6A RNA metabolism.^19^ These findings substantiate the hypothesis that dysregulated m6A modification serves as a pertinent regulatory mechanism influencing the development of ALS.

As a purported m6A reader, RNA binding motif protein X‐linked (RBMX) has been linked to impaired maturation of induced pluripotent stem cell (iPSC)‐derived neurons.^20^ Mechanistically, RBMX engages with RNA polymerase II to govern the alternative splicing of m6A‐modified nascent pre‐mRNA,^21^ a process contingent upon its C terminus Arg‐Gly‐Gly (RGG) motifs within the low‐complexity region.^22^ This gene has previously been posited as a candidate gene associated with X‐linked intellectual disabilities, specifically Shashi syndrome^23^ and Gustavson syndrome.^24^ Additionally, RBMX is implicated in the alternative splicing of the survival motor neuron (SMN) gene in human HEK293 cells by facilitating the inclusion of *SMN2* exon 7,^25^ although any gene dosage effect of *SMN2* on the risk of ALS or disease severity is unclear.^26^ Therefore, the potential role of *RBMX*, which encodes heterogeneous nuclear ribonucleoprotein G (hnRNPG), in the pathogenesis of ALS remains to be elucidated.

To gain a better understanding of the role played by m6A modification and *RBMX* in ALS, we took an integrated approach to investigate their potential pathogenicity. We systematically inspected the mutation landscape of m6A regulator genes using ALS and population‐matched control cohorts. We then analyzed the impact of downregulating *Rbmx* in mouse motor neuron‐like cell line NSC‐34 overexpressing human TDP43 mutant and ALS iPSC‐derived neuron. Finally, the transcriptomic changes induced by *RBMX* knockdown in HEK293T were correlated to the expression profiles of human central nervous system (CNS) cells in primary motor cortex using a single‐cell atlas collected from ALS patients and control subjects.^19^ Overall, the results provided insight into the potential role of *RBMX* and m6A modification in ALS pathophysiology.

## 2 RESULTS

### 2.1 Mutation profiling of m6A regulator genes highlights *RBMX* in ALS pathogenesis

**FIGURE 1:**
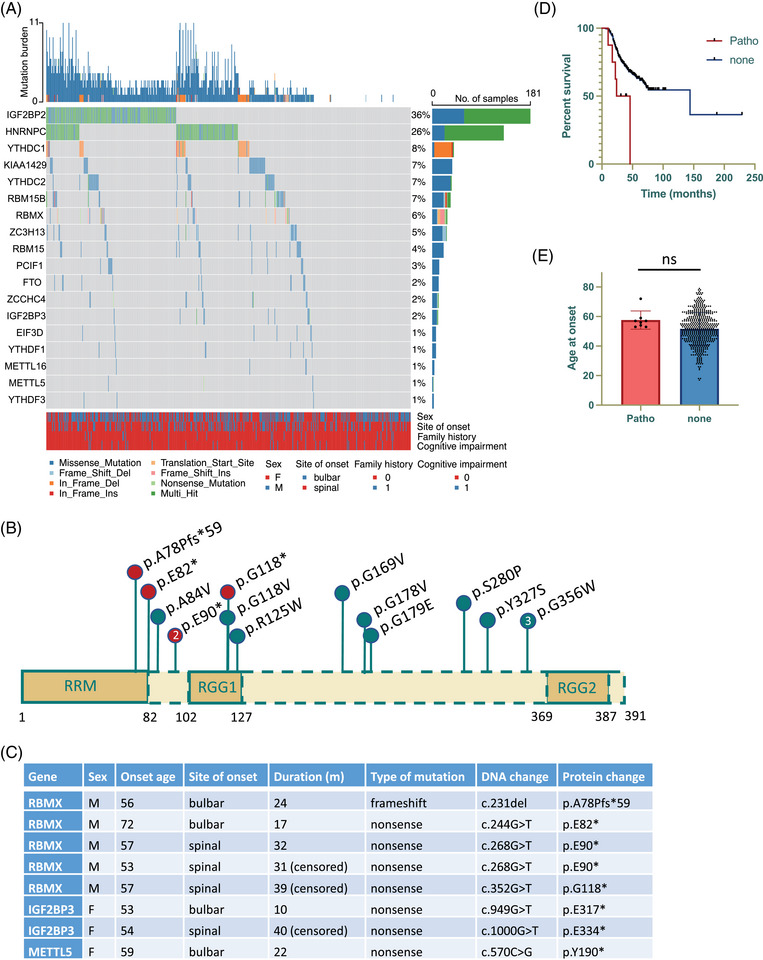
The genetic variants of N6‐methyladenosine (m6A) regulators and their clinical implications in amyotrophic lateral sclerosis (ALS) patients. (A) The top 20 frequently mutated m6A genes in ALS patients. Each column represents one individual patient. (B) Diagram showing the location of the identified RNA binding motif protein X‐linked (*RBMX*) pathogenic mutations (red) and variants of uncertain significance (VUS; blue). RGG, Arg‐Gly‐Gly repeats; RRM, RNA recognition motif. (C) Clinical information of the patients carrying pathogenic m6A variants. (D) Kaplan–Meier survival curves of ALS patients with or without pathogenic m6A mutations. (E) Bar plot comparing the disease onset age between patients with or without pathogenic m6A mutations (ns, not significant).

We first analyzed the whole exome sequencing (WES) datasets to inspect the occurrence of genetic alterations in m6A regulator genes among individuals diagnosed with ALS. The visual representation displayed in Figure 1A revealed that the most frequently mutated genes in patients with ALS were *IGF2BP2* (36%) and *HNRNPC* (26%), followed by *YTHDC1* (8%), *KIAA1429* (7%), *YTHDC2* (7%), *RBM15B* (7%), *RBMX* (6%), and *ZC3H13* (5%). Subsequent burden analysis of these frequently mutated genes demonstrated that only *IGF2BP2* and *RBMX* exhibited a significantly enriched number of variants in patients compared to the control subjects matched by population after Bonferroni correction. To further refine the analysis, we excluded benign and likely benign variants based on American College of Medical Genetics and Genomics (ACMG) guideline. Consequently, we identified a total of 11 missense *RBMX* mutations classified as variants of uncertain significance (VUS), and five pathogenic or likely pathogenic variants (four nonsense mutations and one frameshift mutation), all of which were absent in the control cohort (Figure 1B). In addition to the five pathogenic *RBMX* variants, there were three pathogenic variants identified in other m6A regulator genes (Figure 1C). Patients with pathogenic m6A gene variants manifested poorer clinical outcomes (*p* = 0.0129, Figure 1D), while no significant association was observed with disease onset age (*p* = 0.133, Figure 1E). With regards to the pathogenic *RBMX* variants located on the X chromosome, it is notable that none of the carriers is female or carries any known ALS risk mutations that could otherwise account for the expedited disease progression. Collectively, although it is essential to acknowledge the inherent challenges in drawing definitive conclusions from the limited number of pathogenic variants identified, the findings are indicative that disruption of m6A regulator gene function may potentially influence the clinical outcomes of ALS patients, with *RBMX* emerging as a most prominent candidate.

### 2.2 *Rbmx* downregulation is associated with reduced survival of NSC‐34 cells and transcriptomic changes relevant to ALS pathogenesis

**FIGURE 2:**
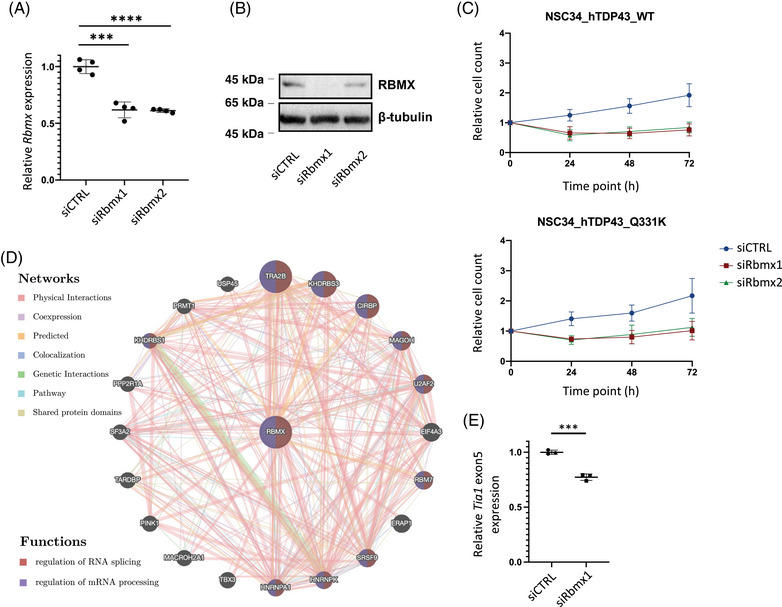
*Rbmx* knockdown in murine motor neurons is associated with cell loss and differential alternative splicing of *Tia1*. (A) Quantitative real‐time reverse‐transcription polymerase chain reaction (qRT‐PCR) validating the expression level of *Rbmx* in NSC‐34 cells transfected with nontargeting scrambled control (siCTRL) and two small interfering RNAs (siRNAs) targeting *Rbmx* (siRbmx1 and siRbmx2). The data were normalized to *Gapdh* and presented as mean ± SEM (***p < 0.001, ****p < 0.0001). (B) Western blot was performed with anti‐RNA binding motif protein X‐linked (RBMX) and anti‐β‐tubulin antibodies. Molecular mass markers are shown on the left in kDa. (C) The expression of *Rbmx* was transiently knocked down in NSC‐34 cells stably transfected with wild type human TDP43 (top) or mutant hTDP43(p.Q331K) (bottom) and cultured for 72 h, with cell number counted every 24 h (*n* = 4). (D) The GeneMANIA network of the top 20 genes most closely connected with *RBMX*; the colors of the edges and nodes represent the types of interaction and the associated functional pathways, respectively. (E) The relative expression level of *Tia1* exon5 in mutant NSC‐34_hTDP43^Q331K^ cells was compared between siCTRL and siRBMX1 conditions (***p < 0.001).

To explore the potential pathogenic roles of *RBMX* in ALS, we utilized murine motor neuron‐like cell line (NSC‐34) with inducible expression of human wild type (WT) or ALS mutant (Q331K) TDP43. The efficacy of *Rbmx* knockdown was confirmed by quantitative real‐time reverse‐transcription polymerase chain reaction (qRT‐PCR; Figure 2A) and Western blot (Figure 2B). We observed reduced cell survival after *Rbmx* knockdown compared to the control small interfering RNA (siRNA; Figure 2C). GeneMANIA, a software tool that employs weighted Gene Ontology (GO) annotations of BP hierarchy,^27^ was used to discern gene interactions that could be of significance to the observed effects of *Rbmx* knockdown in NSC‐34 cells. Among the prominent neighboring genes were the two ALS‐implicated genes, *HNRNPA1* and *TARDBP*, both of which encode RBPs known to recognize m6A modification (Figure 2D). In addition, *TRA2B*, the most correlated gene, has been linked to spinal muscular atrophy (SMA by synergizing with *RBMX* to promote inclusion of *SMN2* exon 7.^28^ These findings suggest that *RBMX* may exert its pathogenic effects in ALS via regulating mRNA metabolism. We thus analyzed the alternative splicing events and differential gene expression following *RBMX* knockdown in human HEK293T cells using the publicly available RNA‐seq dataset (GSE74085).^21^ We observed 1993 differential splicing events (Table S1), along with 2196 significantly upregulated and 1298 downregulated genes [log_2_|FC| > log_2_(1.5), adjusted *p* < 0.05] (Table S2). Among these changes were instances of skipping on *TIA1* exon 5 and differential expression of several known ALS‐associated genes (e.g., *FUS*, *TBK1*, *VCP*, *SQSTM1*, and *KIF5A*) and genes encoding ALS biomarkers, such as neurofilament polypeptides. The *Tia1* exon 5 skipping was confirmed in NSC‐34_hTDP43^Q331K^ cells transfected with siRbmx1 (*p* = 0.0004, Figure 2E). Notably, according to the RM2Target database,^29^ the majority of these differentially expressed genes (DEGs) were affected by perturbation of *METTL3* (45.8% upregulated and 53.6% downregulated) and *METTL14* (37.9% upregulated and 44.7% downregulated) expression, which are the two major components of the heterodimeric N6‐methyltransferase complex involved in m6A modification. Taken together, these findings suggest a potential biological relevance of *RBMX* in the regulation of gene expression in ALS, possibly via an m6A‐dependent mechanism.

### 2.3 Reduced *Rbmx* expression in NSC‐34 cells induces activation of p53 pathway

**FIGURE 3:**
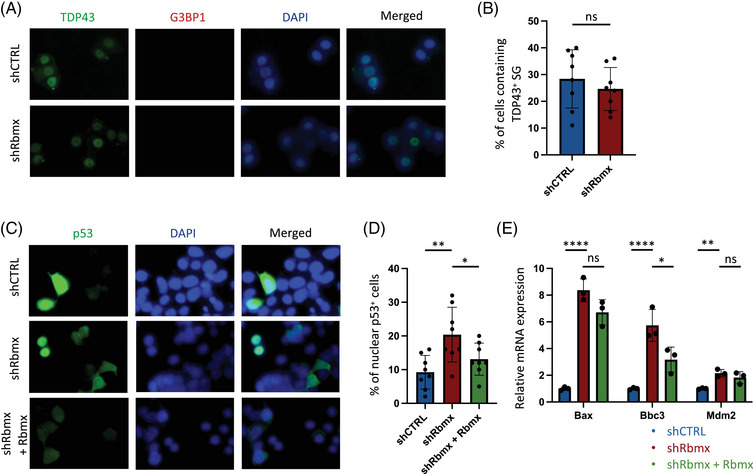
Reduced *Rbmx* expression in NSC‐34 cells induces activation of p53 pathway. (A) NSC‐34 cells stained with anti‐TDP43 and anti‐G3BP1 antibodies in shCTRL and shRbmx conditions, counterstained with DAPI. (B) Quantification of percentage of cells containing TDP43^+^ stress granules (ns, not significant). (C) NSC‐34 cells stained with anti‐p53 antibody in shCTRL, shRbmx, and shRbmx + *Rbmx* conditions, counterstained with DAPI. (D) Quantification of percentage of cells with nuclear p53 positivity (*p < 0.05, **p < 0.01). (E) Relative expression level of *Rbmx*, *Bax*, and *Bbc3* in NSC‐34 cells under shCTRL, shRbmx, or siRbmx + *Rbmx* conditions. The data were normalized to *Gapdh* (*p < 0.05, **p < 0.01, ****p < 0.0001, ns, not significant).

Given that *TIA1* mutations are associated with TDP43 stress granules (SGs) formation through liquid–liquid phase separation (LLPS),^30^ we first examined whether the observed cellular defects in Rbmx‐knockdown cells overlapped with impaired protein homeostasis and SG induction in the absence of exogenous stimuli. We noted that TDP43 was predominantly retained in nuclear fractions in both shRbmx and shCTRL cells, and no G3BP1^+^ SG‐like inclusion was observed in the cytoplasm (Figure 3A). Additionally, there was no difference in the percentage of cells containing TDP43^+^ SGs between shRbmx and shCTRL cells (Figure 3B), indicating that the formation of SG was unlikely the leading cause of the observed cell death. Because loss of RBMX function domain can enhance the p53 response in iPSCs derived from patients of Shashi syndrome,^20^ we next examined the percentage of nuclear p53 in cells by immunostaining (Figure 3C). We found that shRbmx cells demonstrated significantly increased percentage of nuclear p53‐positive cells (*p* = 0.0053), which can be partially rescued by the transient expression of *Rbmx* (*p* = 0.0473, Figure 3D). In accordance with previous findings in iPSCs and U2OS cell line,^20^ reduced Rbmx expression was found associated with increased mRNA levels of some consensus p53 target genes in NSC‐34 cells (Figure 3E).

### 2.4 Reduced *RBMX* expression in iPSC‐derived neurons leads to morphological defects

**FIGURE 4:**
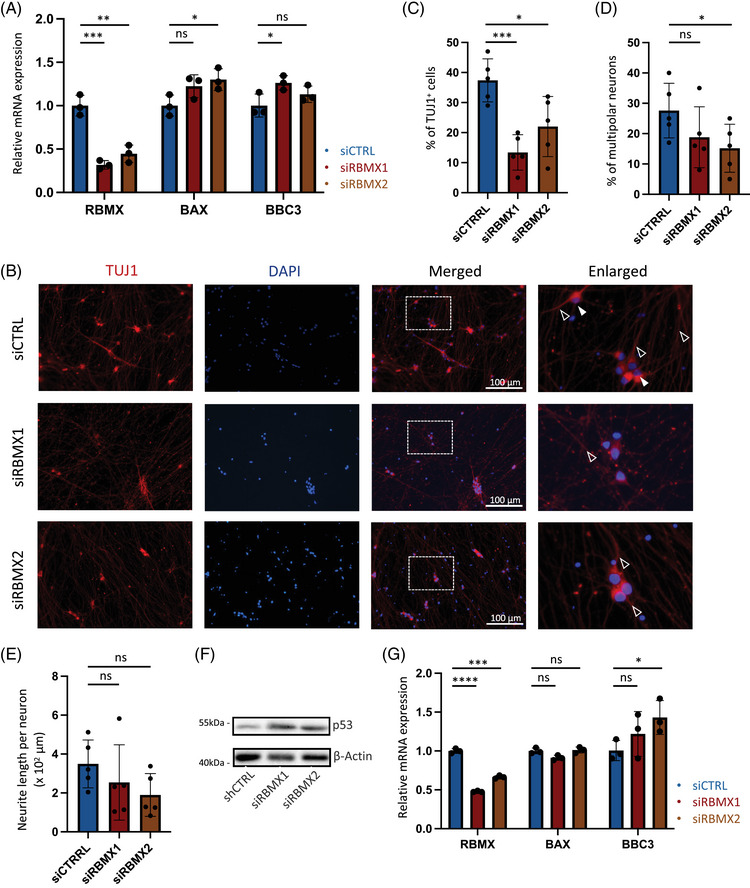
RNA binding motif protein X‐linked (*RBMX*) knockdown in human cell line and amyotrophic lateral sclerosis (ALS) motor neurons induces p53 activation and neuronal defects. (A) Relative expression level of *RBMX*, *BAX*, and *BBC3* in HEK294T treated with siCTRL, siRBMX1, or siRBMX2. The data were normalized to *GAPDH* (*p < 0.05, **p < 0.01, ***p < 0.001, ns, not significant). (B) Staining of induced pluripotent stem cell (iPSC)‐derived motor neurons with anti‐TUJ1 antibody in siCTRL, siRBMX1, and siRBMX2 conditions, counterstained with DAPI. (C) Quantification of percentage of cells with TUJ1 positivity (*p < 0.05, ***p < 0.001). (D) Quantification of percentage of multipolar neurons within TUJ1^+^ cell population (*p < 0.05, ns, not significant). (E) Quantification of neurite length within TUJ1^+^ cell population (ns, not significant). (F) Western blotting of p53 expression in iPSC‐derived motor neuron treated with siCTRL, siRBMX1, or siRBMX2. (G) Relative expression level of *RBMX*, *BAX*, and *BBC3* in iPSC‐derived motor neurons treated with siCTRL, siRBMX1, or siRBMX2. The data were normalized to *GAPDH* (*p < 0.05, ***p < 0.001, ****p < 0.0001, ns, not significant).

To validate the impact of *RBMX* downregulation in human cells, we subsequently evaluated the expression of p53 target genes in response to siRNAs in HEK293T cell line. Consistent with observations in NSC‐34 cells, the knockdown of *RBMX* in HEK293T resulted in elevated expression levels of p53 target genes at the mRNA levels (Figure 4A). Next, we differentiated iPSCs derived from an ALS patient into motor neurons over a 2‐week period using synthetic mRNAs with phosphosite modification,^31^ and modulated *RBMX* expression using siRNAs. The neuronal identity was confirmed by staining for TUJ1, enabling the assessment of morphological changes (Figure 4B). Our findings indicated a significant reduction in the percentage of TUJ1^+^ neurons under siRBMX conditions (Figure 4C), accompanied by a trend of decreased percentage of multipolar neurons (Figure 4D, solid arrows) and reduced neurite length within the surviving TUJ1^+^ population (Figure 4E, hollow arrows). Furthermore, the decay of p53 protein was markedly retarded in siRBMX neurons treated with protein synthesis inhibitor (Figure 4F), and the mRNA level of p53 transcriptional target gene *BBC3* was also enhanced in association with *RBMX* knockdown (Figure 4G). These cumulative results suggest that diminished *RBMX* expression in ALS motor neurons induces morphological aberrations and triggers augmented p53 responses.

### 2.5 The expression of RBMX‐regulated genes in primary motor cortex and their enriched functional pathways

**FIGURE 5:**
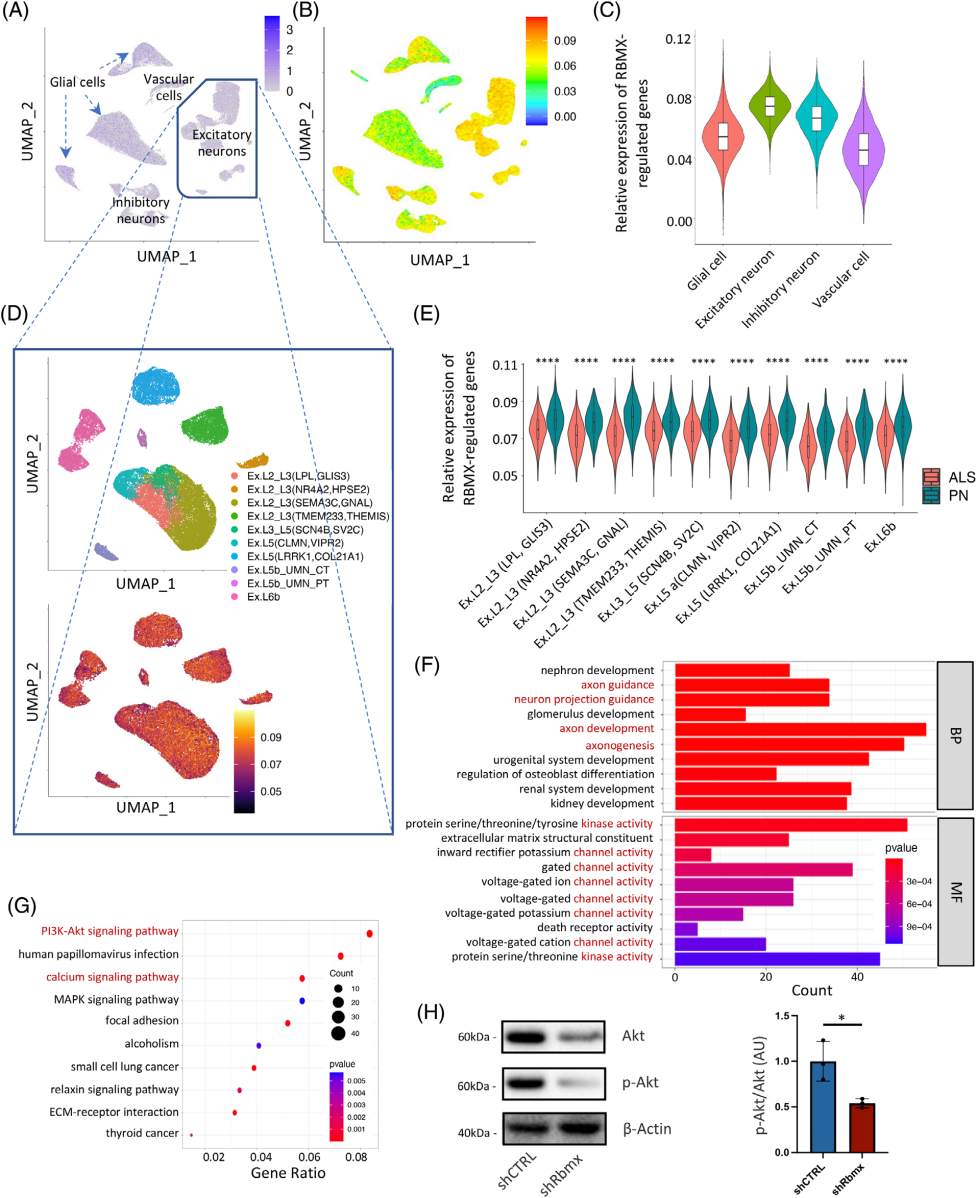
The expression of RNA binding motif protein X‐linked (RBMX)‐regulated genes in primary motor cortex and their enriched functional pathways. (A) Uniform Approximation and Projection (UMAP) feature plot of *RBMX* expression in the characterized cell clusters. (B) UMAP plot of RBMX_score in cell clusters by AddModuleScore function in Seurat package. (C) Violin plot comparing RBMX_score in glial cells, excitatory neurons, inhibitory neurons, and vascular cells. (D) UMAP plots of the identified excitatory neuron subtypes after subclustering excitatory neurons (top) and the corresponding RBMX_score (bottom). (E) Violin plot comparing RBMX_score between amyotrophic lateral sclerosis (ALS) and pathologically normal (PN) subjects across different cellular subtypes of excitatory neuron. (F) Bar plot of Gene Ontology (GO) biologic process (BP) and molecular function (MF) enriched terms colored by *p*‐values. (G) Dot plot of Kyoto Encyclopedia of Genes and Genomes (KEGG) enriched terms colored by *p*‐values. (H) Representative Western blotting and quantification of p‐Akt and Akt expression in NSC‐34 cells (*p < 0.05).

To inspect the molecular functions (MFs) of *RBMX* in human brain, we analyzed the scRNA‐seq dataset of primary motor cortex derived from 17 sporadic ALS patients and 17 pathologically normal (PN) control subjects.^19^ After normalization, scaling, and dimensional reduction, cells were clustered into excitatory neurons, inhibitory neurons, glial cells, and vascular cells according to canonical cell markers in human brain. No noteworthy enrichment of *RBMX* expression was observed in any cell clusters (Figure 5A). The average expression of DEGs resulting from *RBMX* knockdown (log_2_|FC| > 1, adjusted *p* < 0.05) in HEK293T cells was calculated at single‐cell level using the AddModuleScore function, the resulting score of which (RBMX_score) suggested an enriched expression in excitatory neurons (Figure 5B,C). This was of particular interest, as excitatory neurons were known to be the most affected cell populations in ALS pathogenesis. Further dividing the excitatory neurons into subclusters based on the featuring biologic markers obtained from the original study indicated that the enriched RBMX_score was a common feature across different excitatory neuron subtypes (Figure 5D). Interestingly, comparison of the RBMX_score between ALS and PN subjects revealed that the RBMX_score was significantly lower in ALS across these cellular subtypes, including the disease‐associated Betz cells (Ex.L5b_UMN_PT) and L3/L5 long‐range projecting neurons Ex.L3_L5(SCN4B, SV2C; Figure 5E). To explore the potential biologic implications of lower RBMX_score in ALS excitatory neurons, the *RBMX*‐regulated genes correlated with the DEGs in ALS primary motor cortex were selected by Pearson correlation coefficient *r* and the associated *p* value (|*r*| > 0.5, *p* < 0.05) for pathway analyses. The GO MF results indicated that these genes were involved in various protein kinase and gated channel activities, and the BP results suggested that they were related to axongenesis and axon guidance (Figure 5F). In line with these, the Kyoto Encyclopedia of Genes and Genomes (KEGG) analysis suggested that these genes were associated with PI3K‐Akt and calcium signaling pathways (Figure 5G). Given that Akt phosphorylation induced by increased intracellular Ca^2+^ concentration can exert a neuroprotective effect via preventing endoplasmic reticulum stress in ALS neurotoxic models,^32^ we further quantified Akt phosphorylation in NSC‐34 cells. As shown in Figure 5H, downregulating *Rbmx* significantly decreased the phosphorylation levels of Akt (*p* = 0.0231). Collectively, these results indicate that the RBMX‐regulated genes are differentially expressed in ALS primary motor cortex and are likely involved in the pathologic pathways of ALS via affecting excitatory neuron survival.

### 2.6 RBMX‐regulated hub genes and correlated m6A genes in excitatory neurons

**FIGURE 6:**
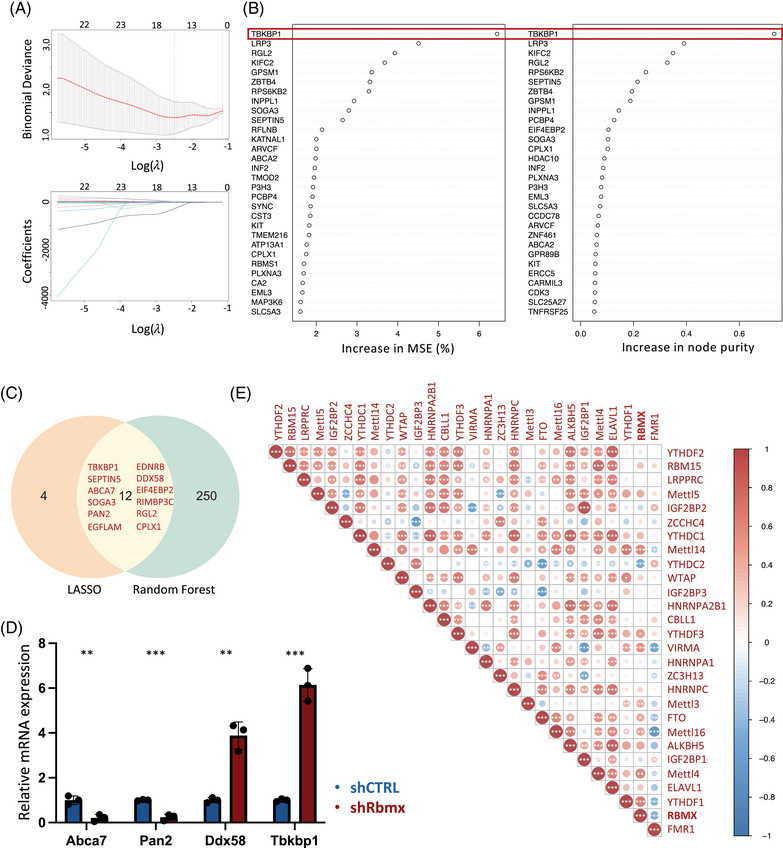
RNA binding motif protein X‐linked (RBMX)‐regulated hub genes and correlation of N6‐methyladenosine (m6A) gene expression in excitatory neurons. (A) Continuous adjustment of potential model parameters yielding the best LASSO regression model (left dashed line) and the simplest model (right dashed line), with the values displayed on the top corresponding to the number of model features at the indicated *λ* value (top); the distribution of regression coefficients for different *λ* values during LASSO regression model construction (bottom). (B) The random forest regression model displaying the genes ranked by the increase in mean square error (MSE) percentage (left) and increase in node purity (right), highlighting *TBKBP1* as the top‐ranked candidate gene. (C) The RBMX‐regulated hub genes identified by intersecting the candidate genes derived from the two regression algorithms, as demonstrated by the Venn plot. (D) Quantitative real‐time reverse‐transcription polymerase chain reaction (qRT‐PCR) validating the expression of the *Rbmx*‐regulated hub genes in NSC‐34_hTDP43^Q331K^ cells transduced with shCTRL or shRbmx. The data were normalized to *Gapdh* (**p < 0.01, ***p < 0.001). (E) Expression correlation heatmap of m6A genes in excitatory neurons by Pearson correlation analysis, with red/blue indicating a positive/negative correlation, respectively (*p < 0.05, **p < 0.01, ***p < 0.001).

To discern the potential pathogenic significance of RBMX‐regulated genes in ALS, we employed machine learning models based on LASSO regression (Figures 6A) and random forest regression (Figures 6B) to analyze the differential expression of these genes between excitatory neurons derived from ALS patients and control subjects. The random forest regression algorithm identified a set of 262 genes, with 12 genes overlapping with the 16 genes identified by least absolute shrinkage and selection operator (LASSO) regression (Figure 6C). Among these genes are several known to be associated with neurodegenerative diseases (*ABCA7* ^33^ and *TBKBP1* ^34^) and m6A‐mediated mRNA metabolism (*PAN2* ^35^ and *DDX58* ^36^). Notably, *TBKBP1* exhibited the highest increase in mean squared error (MSE) and node purity in random forest regression, underscoring its potential relevance to ALS. Subsequently, the expression profiles of these four selected genes were further examined in NSC‐34 cells transduced with shRbmx (Figure 6D). Consistent with the RNA‐seq results obtained from *RBMX* knockdown in HEK293T cells, shRbmx NSC‐34 cells exhibited an upregulation in *Ddx58* expression and a downregulation in *Abca7* and *Pan2* expression. Interestingly, in contrast to the decreased *TBKBP1* expression observed in HEK293T cells, the downregulation of *Rbmx* expression in NSC‐34 cells resulted in a significant increase in *Tbkbp1* expression.

Given that m6A regulators function collaboratively rather than independently,^37^ we proceeded to assess the expression correlation of m6A genes in excitatory neurons. Analysis of their expression in the primary motor cortex unveiled an overall correlated pattern, suggesting overlapping functionalities among these genes (Figure 6E). For examples, the m6A writer *METTL14* exhibited a moderate correlation (*r* > 0.5) with its cofactors *VIRMA* and *WTAP*, while the expression levels of the m6A readers *YTHDC1*, *HNRNPC*, and *HNRNPA2B1*, known for their involvement in mRNA splicing regulation, displayed a strong correlation (*r* > 0.7). Similarly, a close correlation was observed between the expression of *IGF2BP1* and *YTHDF2* and their respective homologues *IGF2BP2* and *YTHDF3*. Remarkably, in the context of *RBMX*, its expression demonstrated a positive correlation (*r* > 0.5) with genes encoding components of the m6A methyltransferase complex (*METTL14, METTL4*) and the reader *YTHDF1*, indicative of a potential synergic association in motor neurons.

## 3 DISCUSSION

In this study, we present evidence substantiating an association between genetic aberrations in *RBMX* and the etiology of ALS. Analysis of mutations revealed a notable prevalence of *RBMX* variants among ALS patients, with null mutations exhibiting a correlation with adverse clinical prognoses. Subsequent experimental investigations employing murine cell line and human iPSC‐derived neurons elucidated that the downregulation of *RBMX* precipitated p53‐mediated apoptosis and induced aberrations in cellular morphology. Furthermore, scrutiny of the scRNA‐seq dataset derived from the human primary motor cortex revealed that genes under the regulatory influence of *RBMX* were predominantly expressed by excitatory neurons and were functionally associated with pathways implicated in ALS pathogenesis.

Impaired RNA metabolism emerges as a prominent feature in the pathogenesis of ALS, often accompanied by mutations in genes encoding RBPs such as *TARDBP* ^38^ and *FUS*.^39^, ^40^ Fibroblasts and iPSCs overexpressing TDP43 display dysregulated m6A modification,^41^ characterized by an increased global abundance associated with a shortened half‐life of m6A‐tagged transcripts in cells.^42^ Correspondingly, TDP43 has been demonstrated to exhibit a preferential binding affinity for m6A‐modified RNA and, in collaboration with the m6A reader protein YTHDF2, mediates neurotoxicity.^43^ In contrast to the observed RNA hypermethylation associated with TDP43, recent findings have revealed a reduction in m6A modification in iPSC‐differentiated neurons derived from *C9orf72* patients, and the restoration of m6A methylation significantly mitigates the accumulation of RNA repeat expansions and subsequent poly‐dipeptide formation.^44^ These provide evidence suggesting that m6A RNA methylation is critically involved in the extensive RNA destabilization implicated in ALS pathophysiology. In support of this, it has recently been reported that the depletion of *Mettl3* in cholinergic neurons can induce ALS‐like phenotype in mice, and that TDP43 is under the epitranscriptomic control of m6A modification,^45^ which further substantiates the involvement of m6A genes in ALS pathogenesis.

Among the currently identified m6A regulators, the genic intolerance scores attributed to *RBMX* indicate a relative vulnerability to loss‐of‐function mutations.^23^ Prior genetic studies based on familial data have identified frameshift and in‐frame deletion mutations in individuals presenting with X‐linked intellectual disability.^23^, ^24^ These findings suggest that mutations affecting *RBMX* constitute a credible pathogenic mechanism aligning with established inheritance patterns. The absence of deleterious *RBMX* mutations in population‐matched controls, alongside its evolutionary conservation, underscores its candidacy as a plausible gene associated with ALS. Furthermore, the exclusive observation of nonsense and frameshift *RBMX* mutations in males is also noteworthy. Given that female carriers may remain asymptomatic, this observation resonates with the reported male predominance in ALS incidence, characterized by a standardized male‐to‐female ratio of 1.35.^46^ Although female *Sod1* transgenic mice have demonstrated delayed disease onset and prolonged lifespan compared to their male counterparts,^47^ studies on *Sod1* mutant rat models have failed to reveal a modulative effect on disease development following gonadectomy,^48^ suggesting that hormonal influences alone may not entirely explain the observed sex preference.^49^ To attain a more comprehensive understanding of *RBMX's* role in sexual dimorphism within ALS and the underlying biological pathways, further investigations employing genetically modified mouse models may be required.

In the in vitro cell models, we conducted gene knockdown experiments to elucidate the role of *RBMX* in the viability of neuronal cells. Consistent with previous report,^20^ NSC‐34 cells and ALS iPSC‐derived motor neuron with reduced *RBMX* expression demonstrated elevated p53 response. The investigation into transcriptional alterations resulting from *RBMX* knockdown in the HEK293T cell line revealed a set of DEGs. To ascertain the predominant cell type expressing these RBMX‐regulated genes in the CNS, we conducted a detailed examination of primary motor cortex scRNA‐seq data. The results revealed that the expression of these genes, as denoted by the RBMX_score, was enriched in excitatory neurons, which aligns with the concept that excitatory neurons are particularly susceptible to cellular defects specific to ALS. Previous investigations have disclosed that excitatory neurons manifest the most pronounced degree of transcriptional dysregulation in ALS,^19^ and notable pathological changes have been observed within Betz cells.^50^ Consistently, the heightened susceptibility to excitotoxicity mediated by disturbed glutamatergic neurotransmission has been recognized as a pivotal pathophysiological characteristic of ALS.^51^, ^52^ It is worth noting that Riluzole is believed to alleviate excitotoxic effects by diminishing presynaptic glutamate release and persistent sodium current.^53^ Hence, prospective functional studies concentrating on excitatory neurons hold the potential to furnish further insights into the pathological roles of *RBMX* in ALS.

In conjunction with the diminished RBMX_score evident in ALS excitatory neurons, the bioinformatic analyses have highlighted a set of si*RBMX*‐affected genes manifesting distinctive expression patterns in ALS, including *TBKBP1* and *DDX58*. Through a comprehensive scrutiny of genome‐wide association studies (GWASs), novel candidate susceptibility loci for frontotemporal dementia (FTD) have been pinpointed within *TBKBP1*.^34^ Mechanistically, TBK1 is recruited by TBKBP1 to the protein kinase C‐theta (PKC‐θ), where it undergoes phosphorylation and activation.^54^ Given that the diminution of TBK1 kinase activity in CNS cells may contribute to neurodegeneration through compromised autophagy,^55^ mitophagy,^56^ endosomal pathway,^57^ and membrane trafficking,^58^ RBMX may participate in ALS pathogenesis by disturbing *TBKBP1* expression. Additionally, TBK1 also plays a role in the RIG‐I (encoded by *DDX58*)‐mediated interferon (IFN) secretion via the IKKε/TBK1/IRF3 pathway, with the m6A sites of *DDX58* deemed indispensable for its maturation.^36^ The accrual of double‐stranded RNA regulated by TDP43 can incite necroptosis through a DDX58‐dependent IFN response.^59^ Collectively, these findings suggest a plausible regulatory role of *RBMX* in the expression of ALS‐implicated genes and pathways, potentially involving m6A‐dependent regulatory mechanisms.

One limitation of the single‐cell sequencing analysis is that the transcriptomic changes induced by *RBMX* downregulation were inferred from immortalized human cell lines. It is also important to recognize that cultured cells undergo distinct transcriptional regulation compared to in vivo settings, and the observed transcriptomic patterns in postmortem tissues may not necessarily reflect the actual pathophysiologic processes in ALS, particularly at early stages. Assessment of the neuronal activity via electrophysiological recordings or calcium imaging could provide valuable functional insights. However, this requires the establishment of in vivo mouse models carrying *RBMX* loss‐of‐function mutations. On the other hand, it is crucial to assess whether male patients carrying loss‐of‐function mutations exhibit downregulated expression of *RBMX* in the CNS. Conducting large‐scale genetic screening could be instrumental in identifying such patients who are accessible for the assessment or capable of providing samples for the construction of iPSC‐derived neurons. Alternatively, given that neuronal–glial cell crosstalk is recognized as a critical factor in the pronounced neurotoxicity elicited by TDP43,^60^ the use of multicellular in vitro models employing motor neurons and microglia derived from CRISPR‐Cas9‐generated iPSCs with *RBMX* knockout could yield additional insights into the pathophysiology of ALS. Furthermore, a comprehensive examination of the effects of m6A reduction, achieved through *METTL3* or *METTL14* knockout, on *RBMX*‐regulated genes in iPSCs may provide direct evidence supporting the pathogenic role of RBMX as an m6A reader in ALS.

Our integrated analyses collectively suggest that *RBMX*‐mediated post‐transcriptional regulation is implicated in ALS pathophysiology. Through our demonstration, we have confirmed that the disruption of *RBMX* expression can induce neuronal defects, with a likely preference for impacting the survival of excitatory motoneurons in the primary motor cortex. Further research in this domain holds the promise of offering significant insights with important implications for the treatment of ALS.

## 4 MATERIALS AND METHODS

### 4.1 Cohort demographics

In total, 508 patients of Chinese ethnicity diagnosed as clinically definite, probable, or probable laboratory‐supported ALS according to El Escorial criteria were included for the mutation analysis (Table S3). The recruitment was taken place at Peking Union Medical College Hospital (PUMCH) between December 2017 and July 2021. Patient follow‐ups were conducted by neurologists in 6‐month intervals via telephone or during patients’ return visits until December 2022. All participants have provided written consent or given permission for a relative to sign on their behalf. Ethical approval for this study was obtained from the PUMCH Research Ethical Boards (No. JS‐2624). The control cohort included 1660 population‐matched healthy individuals from the HuaBiao Project.^61^

### 4.2 Whole‐exome sequencing and variant interpretation

Detailed methodology of WES is as described previously.^61^, ^62^ In brief, DNA was extracted from the peripheral blood using DNA Isolation Kit (Blood DNA Kit V2, #CW2553) and sheared by Bioruptor UCD‐200 (Diagenode). KAPA Library Preparation Kit (Kapa Biosystems, #KR0453) and SureSelect XT2 Target Enrichment System (Agilent) were used for library preparation. DNA libraries were sequenced on the Illumina NovaSeq platform as paired‐end 150‐bp reads in accordance with the manufacturer's specifications. Illumina Sequence Control Software (SCS) was applied to the raw data for variant quality control and downstream analysis. The reads were filtered based on the following criteria: (1) reads with adapter contamination (over 10 nucleotides aligned to the adapter); (2) reads containing more than 10% unidentified nucleotides; (3) the paired reads with single read having more than 50% low quality nucleotides (Phred quality <5). Percentage of reads with average quality >Q30 and GC content distribution were calculated and summarized. High quality reads were subsequently aligned to the UCSC human reference genome sequence (build 37.1 version hg19) using Burrows–Wheeler Alignment tool. Genome Analysis Toolkits (GATK) were used for variant calling according to the GATK Best Practices, with low quality variants filtered based on the following criteria: (1) Quality by depth <2.0; (2) Mapping quality <40.0; (3) Fisher strand >60.0; (4) Mapping quality rank sum test ≤12.5; (5) Read position rank sum ≤8.0. The called variants (call rate >90% and Hardy–Weinberg equilibrium test *p* > 10^−6^) were annotated using ANNOVAR (version: 2016‐05‐1110:54:48‐0700).

The m6A panel used in this study composed genes whose protein products have been implicated as m6A “writer,” “eraser,” or “reader” based on relevant literature sources and the RM2Target database.^29^, ^63^ These include m6A methyltransferase complex components *METTL3*, *METTL 4*, *METTL5*, *METTL14*, *METTL16*, *WTAP*, *RBM15*, *RBM15B, VIRMA* (*KIAA1429*), *ZC3H13*, *ZCCHC4*, *CBLL1* and *PCIF1*; m6A demethylases *FTO* and *ALKBH5*; m6A recognizers *YTHDF1/2/3*, *YTHDC1/2*, *IGF2BP1/2/3*, *LRPPRC*, *ELAVL1*, *FMR1*, *HNRNPC*, *HNRNPA2B1*, *HNRNPA1*, and *RBMX*. The “Maftools” package was used to generate visual representations of the mutation landscape in the ALS cohort.^64^ To enhance the specificity of the mutation profile, variants with a minor allele frequency (MAF) exceeding 0.01 in the gnomAD East Asian population were excluded from the visualization. The pathogenicity of the identified variants was assessed based on the guidelines provided by the ACMG.^65^, ^66^ The *RBMX* variants that met the following criteria were selected for further evaluation: (1) nonsynonymous exonic variants annotated as missense, start–lost, stop–gained, stop–loss, or frameshift mutations; (2) MAF lower than 0.01 in the HuaBiao cohort; (3) significant allelic association with ALS by standard Fisher's exact test. The in silico ensemble prediction tools such as M‐CAP, CADD, and REVEL were used to evaluate the pathogenic potential of these qualifying rare variants. The ALS variants defined as disease‐causing or likely disease‐causing mutations (DM/DM?) according to Human Gene Mutation Database (HGMD) were screened for all patients.

### 4.3 Cell lines

NSC‐34 and HEK293T cells were acquired from Ubigene. Cells were maintained in complete high glucose Dulbecco's modified Eagle medium (DMEM) supplemented with 10% fetal bovine serum (FBS) and 1% penicillin/streptomycin (P/S) solution, cultured in a humidified atmosphere with 5% CO_2_ at 37°C and passaged every 2–3 days. For motor neuron differentiation, NSC‐34 cells were seeded onto collagen‐A‐coated 96‐well plate at a concentration of 4 × 10^3^ cells per well and allowed to adhere overnight. The medium was exchanged to the differentiation medium containing DMEM/Nutrient Mixture F‐12 (DMEM/F‐12, Thermo Fisher, #11320033), 1% FBS (Thermo Fisher, #10099), 1% modified Eagle's medium nonessential amino acids (NEAA, OriCell, #NEAA‐10201‐100), 0.5% P/S and 5 µM all‐trans retinoic acid (atRA, Solarbio, #A9120) 24 h after seeding.

### 4.4 Plasmids construction and generation of stably transduced NSC‐34 cells

NSC‐34 cells stably transduced with human WT or ALS mutant (Q331K) TDP43, and NSC‐34_hTDP43^Q331K^ cells stably transduced with shRbmx were constructed by Ubigene Biosciences Co., Ltd. Specifically, the coding sequence of hTDP43‐EGFP and hTDP43^Q331K^‐EGFP was synthesized and inserted into YOE‐LV001 vector (Ubigene) to get gene overexpression plasmid. The reconstruct plasmid was verified by Sanger sequencing. For lentiviral production, HEK293T cells were cotransfected with lentiviral plasmid carrying WT and mutant hTDP43 using Lentiviral Packaging Kit (Ubigene, #YK‐LVP‐40). The culture medium was collected at 48 h after transfection, lentivirus was titrated by qPCR, and lentivirus particles were stored at −80°C until use. Stable hTDP43‐EGFP and hTDP43^Q331K^‐EGFP gene overexpressing NSC‐34 cells were established using overexpression lentivirus, which were subsequently selected by drug screening with optimal concentration. The expression of WT and mutant hTDP43 was verified by real‐time quantitative PCR (RT‐qPCR). Similarly, shRbmx‐EGFP and shRbmx NSC‐34_hTDP43^Q331K^ cells were constructed and verified by RT‐qPCR (*Rbmx* downregulated by 68.0%).

### 4.5 Transient transfection

siRNAs targeting mouse *Rbmx*, human *RBMX*, and nontargeting scrambled control were purchased from RiboBio Co., Ltd, and the *Rbmx* plasmid was constructed by Ubigene Biosciences Co., Ltd. The siRNA sequences used are as shown in Table S4. The transfection was achieved by using Lipofectamine 3000 (Thermo Fisher Scientific, #L3000001) or Lipofectamine RNA iMax (Thermo Fisher Scientific, #13778100) in accordance with the manufacturer's instructions. For all knockdown transfections, 20 nM siRNA was used.

### 4.6 iPSC and motor neuron differentiation

iPSCs were generated from peripheral blood mononuclear cells collected from a sporadic ALS patient without known ALS‐associated mutation. Reprograming was performed using the CytoTune‐iPS Reprogramming Kit (Thermo Fisher Scientific, #A16517). The cell line was maintained in mTeSR1 medium (Stem Cell Technologies, #85850) at 5% CO_2_, 37°C, and was passaged using ReLeSRTM (Stem Cell Technologies, #100‐0484). Motor neuron differentiation was performed as described previously.^31^ In brief, iPSCs were cotransfected daily with Ngn2 and Olig2 phosphosite‐modified mRNAs for 3 days in medium containing sonic hedgehog (SHH, 100 ng/mL) and N‐[N‐(3,5‐difluorophenacetyl)‐l‐alanyl]‐S‐phenylglycine t‐butyl ester (DAPT, 10 µM). The differentiated cells were dissociated by StemPro Accutase (Thermo Fisher Scientific, #A1110501) and replated to poly‐D‐lysine/laminin‐coated surface at the density of 1 × 10^5^ cells/cm^2^. Cells were maintained in BrainPhys Neuronal Medium (Thermo Fisher Scientific, #05793) until analyses.

### 4.7 Immunofluorescence

Cells were rinsed with phosphate buffered saline (PBS) and fixed with 4% paraformaldehyde (PFA) in PBS for 10 min at room temperature. Afterward, cells were rinsed and blocked with blocking buffer containing 2% bovine serum albumin (BSA) and 0.1% Triton X‐100 for 30 min at room temperature. Cells were incubated overnight at 4°C with primary antibodies diluted in blocking buffer: p53 (ABclonal, #A10610), G3BP1 (ABclonal, #A3968), TDP43 (ABclonal, #A25125), and Tuj1 (CST, #4466S). Next, cells were rinsed with PBS and incubated with secondary antibodies diluted in blocking buffer at room temperature for 1 h: Alexa Fluor 488 donkey anti‐goat (Invitrogen, #A32814), Alexa Fluor 555 goat anti‐mouse (Invitrogen, #A32727). Finally, cells were mounted with Prolong Diamond Antifade Mountant with DAPI (Invitrogen, #P36962), with images captured by Nikon ECLIPSE Ts2R and analyzed by ImageJ. The automated quantification of neurite length was achieved by using the ImageJ plugin, NeuriteTracer, as specified by the original study.^67^

### 4.8 Western blotting

The cells were collected and lysed by gentle scraping in ice‐cold radio immunoprecipitation assay (RIPA) buffer with protease and phosphatase inhibitors. Protein concentrations were measured by a BioPhotometer (Eppendorf), with 30 µg of each sample used for electrophoresis. Protein lysates were denatured at 98°C for 10 min in Laemmli sample buffer, separated by sodium dodecyl sulfate‐polyacrylamide gel electrophoresis (SDS‐PAGE) and transferred to polyvinylidene difluoride (PVDF) membranes. The membranes were blocked with 5% powdered milk in tris buffered saline with tween‐20 (TBST) at 4°C with rocking and incubated overnight with primary antibodies. The following primary antibodies were used: RBMX (Cell Signaling Technology, #14794), p53 (ABclonal, #A10610), Akt (ABclonal, #A17909), p‐Akt (ABclonal, #AP0098), β‐tubulin (Absin, #abs137976), β‐actin (Thermo Fisher Scientific, #EM21002). Membranes were subsequently incubated with horse radish peroxidase (HRP)‐conjugated secondary antibodies (ZSGB Biotech, #ZLI‐9018) and revealed with enhanced chemiluminescence (ECL). The images were visualized and taken using a Molecular Imager Imaging System (Tanon). For p53 assessment, the cells were incubated with 30 µg/mL cycloheximide for 60 min before being subjected to immunoblotting.

### 4.9 Quantitative real‐time PCR

Total RNA was extracted using the TRI Reagen (Invitrogen, #AM9738) according to the manufacturer's protocol, and treated with DNaseI using the PerfeCTa DNaseI kit (Quantabio, #95150‐100). The complementary DNA was then prepared using the qScript cDNA Synthesis Kit (Quantabio, #95048‐025). RT‐qPCR was performed using the SYBR Premix Ex Taq (Takara, #4472903) on a QuantStudio 12K Flex Real‐Time PCR System (Life Technologies). The results were *Gapdh*‐normalized, and the quantification of the mRNAs relative expression was performed using the 2^−∆∆Ct^ method. The primers used are as shown in Table S4.

### 4.10 Analysis of bulk mRNA sequencing data

The differential gene expression resulting from *RBMX* knockdown in HEK293T cells was obtained from GEO database (GSE74085).^21^ Differential expression levels of genes were analyzed by using DEseq2.^68^ The fold change (FC) in gene expression levels was presented as log_2_[Counts_(siRBMX)_/Counts_(siCTRL)_]. DEGs were selected using the threshold log_2_(|FC|) > 1 and adjusted *p*‐value <0.05. Alternative splicing analysis was performed by replicate multivariate analysis of transcript splicing (rMATs) v3.2.5 software using default settings.^69^ Significant alternative splicing events were called at a false discovery rate (FDR) cutoff of 0.05.

### 4.11 Analysis of single‐cell mRNA sequencing data

The Seurat R toolkit (version 4.2.1)^70^ was used for analyzing the gene–barcode matrix, which was made publicly available by Pineda et al. (GSE174332).^19^ Low quality cells (<50 genes/cell, <3 cells per genes and >7% mitochondrial genes) were excluded for downstream analysis. Principal component analysis (PCA) was performed on the top 2000 most variable genes, and the first 15 PCs were used for k‐means clustering and construction of k‐nearest neighbor graph, with the clustering resolution parameter set at 0.5. The clustered cells were visualized using Uniform Approximation and Projection (UMAP) method. The cells were functionally annotated based on canonical CNS cell markers and the curated set of cell type‐specific markers used in the original manuscript. Differential gene testing was performed via FindMarkers function using the default nonparametric Wilcoxon rank sum test. The RBMX_score was calculated by AddModuleScore function in Seurat with default parameters. Pearson correlation analysis was performed to select the RBMX‐regulated genes that correlate to the genes differentially expressed in ALS samples (|*r*| > 0.5, adjusted *p*‐value <0.05) for functional enrichment analysis, with the top enriched terms displayed in ascending order of *p*‐value. Random forest regression was performed via the R package “randomForestSRC,” with the number of feature trees set at 100 and the number of random splits at one. LASSO regression was performed via the R package “glmnet,” with 100 times cross validation. The expression correlation between m6A genes was calculated and visualized using ggcorrplot package in R.

### 4.12 Statistical analysis

GraphPad Prism (version 7.0) and R (version 4.2.2) were used for all statistical analyses. Student's *t*‐tests were applied to compare between two groups. Results were presented in the form of means ± standard deviation (SD) with at least three biological replicates. Fisher's exact test was used to compare the allele frequency in the ALS cohorts versus the control HuaBiao cohort. The aggregate association of rare variants (MAF < 0.01) with ALS was evaluated using sequence kernel association test (SKAT) analysis.^71^ The adjusted *p*‐values were calculated with the formula: *p* = 1 − (1 − *p*)*^n^*, where *p* is the *p*‐value obtained from the test and *n* is the number of tests completed. Survival analysis was conducted using log‐rank Mantel–Cox test. Kaplan–Meier curves were used to graphically display the time interval between disease onset and death or censoring. All statistical tests were two‐tailed, with a nominal or Bonferroni‐corrected significance threshold set to 0.05.

## AUTHOR CONTRIBUTIONS

Study conception and design, Di He; performed experiment and data analysis, Di He, Xinyi He, and Liyang Liu; clinical data collection, Liying Cui, Mingsheng Liu, Xunzhe Yang, and Dongchao Shen; Supervision, Liying Cui and Xue Zhang; manuscript drafting, Di He. All authors contributed to data interpretation and manuscript revision. All authors read and approved the final manuscript.

## CONFLICT OF INTEREST STATEMENT

The authors declare no conflicts of interest.

## ETHICS STATEMENT

Ethical approval for this study was obtained from the PUMCH Research Ethical Boards (I‐23PJ1218). All participants have provided written consent or given permission for a relative to sign on their behalf.

## Supporting information

Appendices
Table S1 Differential splicing events resulting from *RBMX* knockdown in HEK293T cell.
Table S2 Differential gene expression resulting from *RBMX*, *METTL3* and *METTL14* knockdown, respectively.
Table S3 Demographic features of the ALS cohort.
Table S4 Primers and siRNA sequences.

## Data Availability

Data generated and analyzed during this study are included in the Supporting Information files. The *RBMX*‐knockdown RNA‐seq data and human primary motor cortex scRNA‐seq data were obtained from GEO database (GSE74085 and GSE174332). The variants identified in ALS patients analyzed during this study are deposited in the Genome Variation Map (GVM) in National Genomics Data Center, Beijing Institute of Genomics, Chinese Academy of Sciences and China National Center for Bioinformation, under accession number GVM000588. Individual level WES sequencing data are deposited in the Scientific Data Center for USTC under accession number 38458.11.USTC.ovJYiq0U, which are available from the corresponding authors on reasonable request.
